# Pediatric Chagas disease in the non-endemic area of Madrid: A fifteen-year review (2004–2018)

**DOI:** 10.1371/journal.pntd.0010232

**Published:** 2022-02-24

**Authors:** Luz Yadira Bravo-Gallego, Laura Francisco-González, Álvaro Vázquez-Pérez, Milagros García-López Hortelano, Rogelio López Vélez, Luis Ignacio González-Granado, Mar Santos, Cristina Epalza, Ana Belén Jiménez, María José Cilleruelo, Sara Guillén, Tania Fernández, Iciar Olabarrieta, María Flores-Chavez, José Tomás Ramos Amador, María Isabel González-Tomé

**Affiliations:** 1 Department of Pediatrics, Hospital La Paz, Madrid, Spain; 2 Department of Pediatrics, Hospital Clínico San Carlos, Complutense University of Madrid, Madrid, Spain; 3 Pediatric Infectious Diseases Unit, Hospital La Paz, Madrid, Spain; 4 National Referral Unit Of Tropical Diseases, Infectious Diseases Department, Hospital Universitario Ramón y Cajal, Madrid, Spain; 5 Primary Immunodeficiencies Unit, Department of Pediatrics, Hospital 12 Octubre; Research Institute Hospital 12 octubre (i+12); Complutense University School of Medicine, Madrid, Spain; 6 Pediatric Infectious Diseases Unit, Hospital Gregorio Marañón, Madrid, Spain; 7 Pediatric Infectious Diseases Unit, Hospital 12 de Octubre, Madrid, Spain; 8 Department of Pediatrics, Hospital Fundación Jiménez Díaz, Madrid, Spain; 9 Department of Pediatrics, Hospital Puerta de Hierro, Majadahonda, Spain; 10 Department of Pediatrics, Hospital Universitario de Getafe, Getafe, Spain; 11 Department of Pediatrics, Hospital Militar Gomez Ulla, Madrid, Spain; 12 Department of Pediatrics, Hospital Severo Ochoa, Leganés, Spain; 13 Department of Parasitology, National Microbiology Centre (CNM), Instituto de Salud Carlos III, Madrid, Spain; 14 Department of Public and Maternal and Child Health of the Complutense University of Madrid. Hospital Clínico San Carlos. Instituto de investigación Sanitaria del Hospital Clínico San Carlos (IdISSC), CIBERINFEC, Madrid, Spain; Instituto de Ciências Biológicas, Universidade Federal de Minas Gerais, BRAZIL

## Abstract

**Background:**

Chagas disease (CD) has become an emerging global health problem in association with the immigration of individuals from endemic areas (in LatinAmerica) to other countries.Spain is the country in Europe with the highest number of CD cases. Concerning pediatric CD, treatment is not only better tolerated by younger children but also has greater cure possibilities. The aim of this study was to describe clinical and epidemiological aspects of CD in a pediatric population diagnosed of 10 hospitals in the Community of Madrid during the 2004–2018 period, as well as the safety and efficacy of CD treatment on this population.

**Methodology/Principal findings:**

A multicenter, retrospective, descriptive study was conducted. The studied population included all identified children under the age of 18 with a diagnosis of CD. Diagnosis was performed with a positive parasitological test (with subsequent confirmation) or confirmed persistence of positive serology beyond 9 months, for children younger than one year-old, and with two different positive serological tests, for children older than one.

Fifty-one children were included (59% male; 50.9% born in Spain). All mothers were from Latin America. The median age at diagnosis was 0.7 months for those under one year of age, and 11.08 years for those older than one year-old. Only one case presented a symptomatic course (hydrops faetalis, haemodynamic instability at birth, ascites, anaemia). For 94% treatment was completed. Considering patients who received benznidazole (47), AE were recorded in 48,9%. Among the 32 patients older than one year-old treated with benznidazole, 18 (56.25%) had adverse events whereas in the 15 under one year, 5(33,3%) did. Eigtheen (78.2%) of the patients with benznidazole AE were older than one year-old(median age 11.4 years). Of the patients treated with nifurtimox (9), AE were reported in 3 cases (33,3%).

Cure was confirmed in 80% of the children under one year-old vs 4.3% in those older (p<0.001). Loss to follow- up occurred in 35.3% of patients.

**Conclusions/Significances:**

Screening programs of CD since birth allow early diagnosis and treatment, with a significantly higher cure rate in children treated before one year of age, with lower incidence of adverse events. The high proportion of patients lost to follow-up in this vulnerable population is of concern.

## Author summary

Chagas disease, caused by the protozoan *Trypanosoma cruzi*, has traditionally been confined to endemic locations in South America (with vectorial transmission), but is nowadays emerging in Europe, mainly in Spain, due to immigration.

A series of characteristics of this disease condition its management. It is usually asymptomatic (especially in early stages), it can be transmitted from mothers to child (vertical transmission) and treatment is more effective at a younger age or during the early stages of infection. For these reasons early diagnosis and treatment of infected children is a priority. To date, there are few publications on pediatric CD series in non-endemic regions. In this paper we describe the epidemiological and clinical characteristics of 51 children diagnosed with pediatric CD in a non-endemic area, as well as the the safety and efficacy of treatment in this patient population.

We found that most patients were asymptomatic at diagnosis. The cure percentage was clearly higher when they were treated before one year-old (80% vs 4.3% in children older than one).Adverse events to treatment have occurred especially in older children, being frequent in this age group (median age 11 years).

The high proportion of patients lost to follow-up that occurs in this vulnerable population is also noteworthy (35.3% in our case). We highlight, as a conclusion of our study, the need of carrying out systematic screening for this disease (women of childbearing age, children of mothers with positive serology) to reduce the rate of vertical transmission and to achieve higher cure rates and better tolerance to treatment.

## Introduction

Chagas disease (CD), caused by the protozoan parasite *Trypanosoma cruzi*, was listed in 2010 by the World Health Organization (WHO) [1] as one of the neglected tropical diseases (NTDs).

However, 6 to7 million people are estimated to be infected around the world [2]. Due to migratory flows in recent decades, CD has shifted from being confined to endemic areas in Latin America, to being a disease that can be diagnosed worldwide.

Traditionally, in endemic areas, infection occured mainly through vector-transmission. Successful results were obtained through the action taken by countries to control vector and transfusion transmission of the parasite. These initiatives led to substantial reductions in these ways of transmission [2]. Vertical (mother-to-child) transmision is the main transmission route in non-endemic countries [3,4,5].

In Europe, Spain is the country with the highest number of CD cases [6]. Spanish guidelines [7] recommend the screening for CD in pregnant women who come from endemic countries, in children born to infected mothers (ideally from birth) and of all children coming from endemic areas, regardless of their age. For the diagnosis of congenital CD in children born to infected mothers, these guidelines recommend preforming: Polymerase Chain-Reaction (PCR) and /or microhaematocrit detection at birth repetead in a second determination, and a serological determination at 9 months of age or later. Nowadays, although this screening is recommended and increasingly widespread it is not universal, so undiagnosed pediatric cases are still ocurring.

CD is usually asymptomatic during childhood, as well as in adulthood. The disease has an acute and a chronic phase. Following the acute phase, most infected people enter a prolonged asymptomatic phase of the disease (called “chronic indeterminate”). Late complications, classically associated with CD, occur in the chronic phase, “chronic determinate”, in approximately one third of the patients [8]. The outcome in children depends on the age at treatment initiation. Treatment is not only better tolerated in younger children but also the possibilities of cure are greater.

There are two approved drugs for CD: benznidazole (of choice) and nifurtimox (alternative treatment). Although there are no randomized clinical trials that compare benznidazole and nifurtimox, benznidazole is generally preferred due to its better tolerability and tissue penetration, as well as its possible higher effectiveness. [9]

There are few publications on pediatric CD series in non endemic regions [10,11]. Children with CD are especially vulnerable, due not only to the high pathogenic potential of the disease but also to their frequent involvement in challenging socioeconomic situations, including migration. [12]

The present study had two principal aims. Firstly, to describe clinical and epidemiological characteristics of CD in a pediatric population diagnosed in 10 hospitals in the Community of Madrid, Spain. Secondly, to describe the safety and effectiveness of the treatment on this pediatric population.

## Patients and methods

### Ethics statement

The study was reviewed and approved by the Ethics Committee of the Hospital Universitario Ramón y Cajal (2014).

Informed consent was not requested from patients as it was a retrospective, observational, risk-free study for patients whose identities were anonymized, in compliance with current legislation.

A multicenter, retrospective, descriptive study was conducted in 10 public hospitals of the Community of Madrid.

### Inclusion criteria

All patients under 18 years of age diagnosed with CD in the participating hospitals, between 2004 and 2018. Patients diagnosed outside of the Community of Madrid were excluded.

### Diagnostic criteria

- Children during the first year of their life: The presence of a positive parasitological test, with direct visualization -microhaematocrit- or more frecuently with molecular biology methods-polymerase chain reaction (PCR)-, with subsequent confirmation, and / or confirmed persistence of positive serology beyond nine months of life (without decline of antibody titers).

We considered these diagnostic criteria for this age group because during the first year of life, a positive serological test can result from the placental transfer of maternal antibodies and because parasitological methods are adequate for the diagnosis of congenital disease, due to the high levels of parasitemia [13,14].

- After one year-old, the diagnosis was undertaken using two different serological tests for CD (enzyme-linked immunosorbent assay, chemiluminescence analysis or immunochromatograpy, depending on the hospital). In case of disagreement between these two tests, for the diagnosis, a third determination was considered necessary, with a different technique (indirect immunofluorescence).

Vertical transmission route was considered in those children that were born in Spain and whose diagnosis was made in the first year of life (followed from birth) and also for those who were born in Spain and did not have a travel history to an endemic area nor blood transfussion, regardless of age at diagnosis.

Details of adverse events (AE) to treatment were not uniformly collected in medical records. We have clasiffied them as **mild**, when symptoms did not interfere with the patient’s daily activity or did not require symptomatic treatment; **moderate**, when clinical manifestations needed treatment or interruption/change of the CD treatment and **severe,** when clinical manifestations entailed vital risk or produced sequels.

Treatment response, was defined as cured when a serological test became negative during the follow-up (confirmed with a second determination) [7].

### Statistical analysis

was performed by describing qualitative variables with absolute numbers and percentages, and quantitative variables with mean and standard deviation, or with median and interquartile range, if the variables did not follow a normal distribution. Statistical significance was set at 0.05 for all tests performed. The statistical analysis was done with SPSS v.25 program (IBM SPSS Inc., Chicago, IL).

## Results

Fifty-one cases of pediatric CD were identified (59% male, 41% female). Twenty-six (50,9%) were born in Spain and twenty-five (49%) came from endemic areas. All the mothers had Latin American origins: 94% (n = 48) came from Bolivia, 2%(n = 1) from México, 2%(n = 1) from Honduras and 2%(n = 1) from Paraguay. None of them had received treatment for CD prior to their children’s diagnosis, despite that 5 (10%) were aware of the diagnosis previously.

The median age at diagnosis was 103.5 months (IQR: 1,4–167). They ranged in age from 0 to 17 years-old. Sixteen cases (31.4%) were diagnosed under one year of age, the median age of diagnosis in this group was 0.7 months (IQR 0.09–1.54). For those older than one year-old (n = 35, 68.6%), the median age of diagnosis was 11.08 years (IQR: 8.14–14.7). The results broken down by age group (under 1 year-old vs over one year of age, are detailed in **Table 1**)

**Table 1:** Comparative table regarding age (<1 year-old vs > 1 year-old).

		Children < 1 year-old. N/total (%)	Children ≥1 year-old. N/total (%)	Total
**N**		16/51 (31,4)	35/51 (68,6)	51
**Median age at diagnosis**		0.7 months (IQR 0.09–1.54)	11.08 y.o. (IQR 8.14–14.7)	--
**Diagnostic procedures**		PCR: 15/16 (93,7)	PCR + Serology: 19/35 (54,3)	34/51
		Serology: 1/16 (6,25)	Only Serology: 16/35 (45,7)	17/51
**Treatment completed**		15/16 (93,7)	33/35 (91,4)	48/51
**Treatment drugs**	**Benznidazole**	14/15 (93,3)	25/33 (75,7)	39/48
	**Nifurtimox**	0/15 (0)	1/33 (3,03)	1/48
	**Benznidazole followed by nifurtimox**	1/15 (6,6)	7/33 (21,2)	8/48
**Treatment adverse events**	**Benznidazole**	5/15 (33,3)	18/32 (78,26)	23/47
	**Nifurtimox**	0/0(0)	3/9 (33,3)	3/9
**Loss to follow-up**	**Total**	6/16 (37,5)	12/35 (34,3)	18/51
	Before treatment	0/16 (0)	2/35 (5,7)	2/51
	During treatment	1/16 (6,2)	0/35 (0)	1/51
	After treatment	5/16 (3,1)	10/35 (28,5)	15/51
**Patients who continued follow up**	**Total**	10/16 (62,5)	23/35 (65,7)	33/51
	Cured	8/10 (80)	1/23 (4,3)	9/33
	Decreasing serological titers	1/10 (10)	10/23 (43,4)	11/33
	Serology remains positive	1/10 (10)	12/23 (52,2)	13/33
**Born in Spain**		16/26 (61,5)	10/35 (28,5)	26/51

Vertical transmission, as defined in the Patients and Methods section, was considered in 26 cases (50.9%). Of these Spanish-born cases, follow-up from birth was performed in 61.5% (16/26). The other 10 (38,4%) were missed oportunities of early diagnosis (diagnosed at more than one year of age). They all (10) were born before 2013, the year of publication of Spanish guidelines for the diagnosis and management of pregnant women and children with Chagas disease [7]. None of these ten patients had a history of travel to endemic areas nor previous blood transfussions either.Their diagnosis (10/26) were reached because they were referred when the mothers were diagnosed with Chagas disease.

In the remaining 25, the route of transmission was not determined, since the children were not born in Spain, and therefore whether infection occured through vertical or vectorial transmission could not be ascertained.

### Clinical data

The reasons for consultation / follow-up of pediatric patients were: mother with positive serology after pregnancy in 22 patients (43%), positive maternal serology during pregnancy in 12 (23.5%), screening of immigrant population from endemic areas in 8 patients (15.6%), mother with positive serology before pregnancy in 5 (10%), and adoption in the remaining 4 (7.8%).

The clinical presentation was asymptomatic in 50 cases (98%), and symptomatic in only one case, which was diagnosed in the acute phase (hydrops fetalis), previously reported [15]. This child had a case of symptomatic congenital Chagas disease (hydrops fetalis, ascites, haemodynamic instability, anaemia) and required admission to the neonatal intensive care unit. The patient had a positive PCR result at birth, and responded favourably to a 60-day course of treatment with benznidazole with resolution of the symptoms.

### Diagnostic testing

In patients younger than one year-old, followed from birth (n = 16 15 cases (94%) were diagnosed by PCR (associated with microhematocrit in two of them), confirmed with a positive serology beyond 9 months of age, while for the remaining case (6%) PCR was not performed at birth, being diagnosed at 10 months, by persistence of positive serology, with no decrease in antibodie titers. In patients older than one year (n = 35), the diagnosis was made with serology for all cases (two determinations, different techniques), associated with positive PCR in 19 (54.3%).

### Treatment and adverse events (AE)

The treatment was initiated for 49 patients (two were lost to follow-up before treatment) and completed by 48 (one patient lost in follow up during the treatment). Among the 48 patients who completed the treatment: 47 were initially treated with benznidazole. One patient was treated with nifurtimox from the beginning, due to a benznidazole shortage problem.

Considering patients who received benznidazole (47), AE were signaled in 23 cases (48,9%). In 4,3% (1/23) the AE were **severe** (DRESS Syndrome), in 56,5% (13/23) considered as **moderate** (medication had to be temporarily discontinued or changed) and in 39,1% (9/23) were **mild**. The patient with the severe AE, was a 15-year-old girl, who presented a clinical picture compatible with DRESS syndrome, 9 days after starting treatment with benznidazole. She required hospital admission and treatment with corticosteroids and antibiotic therapy. Subsequently, sensitization to benznidazole was confirmed, with a lymphoblastic transformation test. She presented good evolution after withdrawal of benznidazole, and later completed uneventfully the treatment with nifurtimox.

Of patients with moderate AE (13): for 5 of them the treatment had to be temporarily discontinued, waiting for an improvement of the AE or to start symptomatic treatment, (60% rash, 40% neutropenia) and 8 cases were initially treated with benznidazole and later on changed to nifurtimox, due to AE (rash 50%, neutropenia 25%, both rash+neutropenia 25%).

Regarding benznidazole AE: 14 (60.8%) were cutaneous (rash), 9 (39,1%) hematologic (anemia, neutropenia, or eosinophilia), and gastrointestinal symptoms (abdominal pain and nausea) were described in 4 (17,4%) cases. Eigtheen (78.2%) of patients with benznidazole AE were older than one year-old (median age 11.4 years). Among the 32 patients older than one treated with benznidazole, 18 (56.25%) had adverse events whereas 5(33,3%) did for the 15 for under one year-old.

Of the patients treated with nifurtimox (9), AE were reported in 3 cases (33,3%), all of them older than one year-old. Two of them presented rash and one insomnia, which was attributed to treatment. The only patient treated up front with nifurtimox had no AE.

### Follow-up

Eigtheen (18/51) patients (35,3%), were lost to follow-up: in 2 cases (4%) before starting treatment (because they returned to their country of origin), in 1 (2%) during treatment (as he moved to another Spanish autonomous community). The remaining 15 (29,3%) patients were lost after having completed the treatment.

For the patients who completed the treatment and continued on follow-up (n = 33): 24 (47%) continue to be monitored (as the serology has not yet been negative), while 9 (17.6%) have been deemed cured and discharged.

Cure (negative serology in at least two determinations) was observed in 80% of patients diagnosed when they were younger than one year-old (8/10), compared to 4.3% of those older than one (1/23), being this difference statistically significant (p <0.0001).

From the 24 patients who had positive serology at the last follow-up visit: 11 (45.8%) had decreasing titers (only 1 of them started treatment within the first year of life) and 13 (54.16%) did not show a decrease in antibody titers (12 of them older than one year-old at diagnosis).

**Fig 1:**
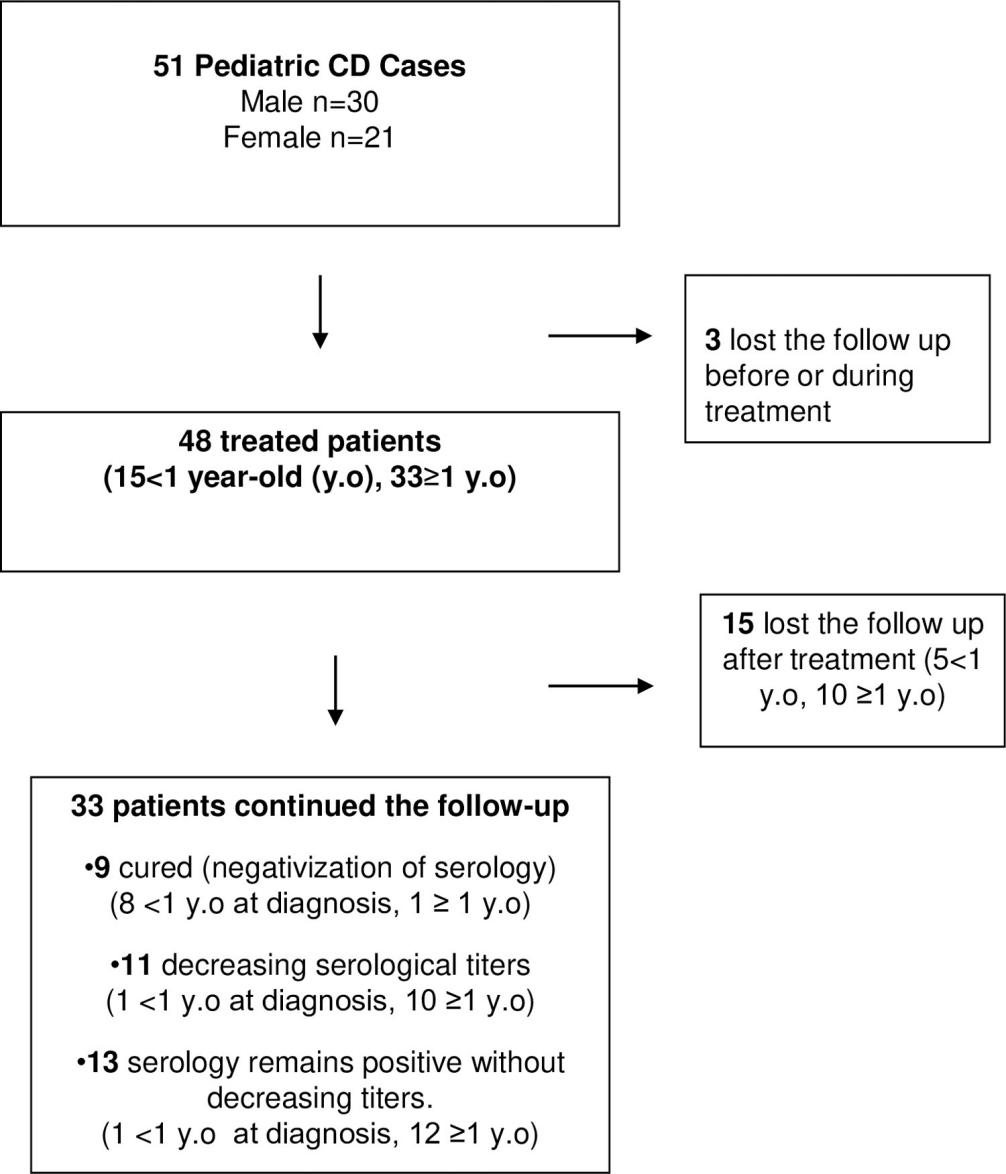
Patient flow chart of the study.

The patient flow chart of the study is detailed in **Fig 1**

For all patients who had a positive PCR, it became negative after treatment. The mean time at which the PCR was negative (after the end of treatment) was 4.5 months (Range: 0.9–15.9). The average follow-up time of cured patients (time until negative serology) was 20.1 months (Range 8–57).

The detailed information of the 33 patients who completed the treatment and continued follow-up is in **Table 2**

**Table 2:** Age at the start of treatment, time of follow up and outcome in the 33 patients that continued follow-up.

	Age at the start of treatment (months)	Follow-up time (months)	Outcome (Serology)
1	0.1	22	Cured
2	0.3	36	Remains positive
3	0.56	19	Cured
4	0.59	15	Cured
5	0.76	31	Decreasing titers
6	1.48	12	Cured
7	1.77	8	Cured
8	2.89	11	Cured
9	2.89	12	Cured
10	7.82	25	Cured
11	41.03	103	Decreasing titers
12	41.92	36	Remains positive
13	55.16	42	Decreasing titers
14	90.22	168	Decreasing titers
15	97.74	57	Remains positive
16	104.28	72	Remains positive
17	105.46	18	Decreasing titers
18	125.37	99	Remains positive
19	122.84	22	Decreasing titers
20	130.79	57	Cured
21	133.19	32	Remains positive
22	137	72	Remains positive
23	147.42	45	Decreasing titers
24	149.36	36	Remains positive
25	165.55	65	Decreasing titers
26	166.08	92	Remains positive
27	177.81	14	Remains positive
28	176.79	72	Decreasing titers
29	191.51	26	Remains positive
30	192.99	146	Decreasing titers
31	200.18	102	Decreasing titers
32	201.92	14	Remains positive
33	202.05	29	Remains positive

The average follow-up time of this 33 patients was 57.8 months (Range 8–168), median time 36 months (IQR 18.5–72).

## Discussion

To our knowledge, we present one of the largest pediatric CD series diagnosed, treated and followed in a non-endemic area, regardless of their origin. As in other published series [10,11], Bolivia was the most frequent country of origin of the cases or of their mothers in our study.

As main findings of our study, we highlight the need to perform screening programs of CD to allow early diagnosis and treatment, as most patients were asymptomatic. It is also important to highlight the significantly higher cure rate in early treated chidren (younger than one year-old), with lower incidence of adverse events to treatment also for this group. The high rate of loss to follow-up in this vulnerable population is also noteworthy.

It is important to carry out children screening from birth when the mothers have a previous positive serology (33.5% of patients in our series) [7], as well as the need to study the previous children of these mothers (in our series, 43% of patients were so diagnosed). Despite this, of the patients in the series born in Spain, 38,4% had not been followed from birth, maybe because this follow-up recommendations (including PCR and /or microhaematocrit detection at birth and 1 month later, and a serological determination at 9 months of age or later) were not published in our country until 2013[7], and all of them were born before this year. Although there were already recommendations before, there was perhaps less awareness. Even today this screening is not done systematically in all hospitals, even though it is becoming more widespread.

Furthermore, a good strategy to avoid disease underdiagnosis would be to screen people from endemic areas in primary care, particularly women of childbearing age. It is of paramount importance to treat the disease prior to pregnancy, since it might reduce or even prevent the rate of congenital transmission [7,16,17]. In our work, in 10% of the cases, even though the mothers knew the diagnosis before gestation, they had not been treated.

Another group to which special attention should be paid are immigrant population and internationally adopted children from Latin America [18]. 7% of the patients in our case series were diagnosed by screening from the intercountry adoption consultation.

CD is usually asymptomatic when there is vertical transmission, or other transmission route during childhood. In our case, 98% of the patients did not present suggestive symptoms of the disease at diagnosis

For patients with congenital infection, presenting with symptoms at birth, these may be variable and usually involve several organs and systems, as in the case of the only symptomatic patient in our series [15].

In other published series of pediatric-age patients in non-endemic areas, most cases have also been asymptomatic [10,11,19].

In their work, Fumadó et al [10] describe a series of 22 cases, 5 of congenital transmission, all asymptomatic, and mostly from Bolivian origin. Rodriguez-Guerineau [11] et al, report 45 cases of Chagas disease diagnosed or treated in Spain and Switzerland, with only one symptomatic case, with gastrointestinal complications in the chronic phase of the disease (megaesophagus). In our series there were no cases of megaesophagus or megacolon.

In their recent paper, Simón et al [19] describe 40 cases of Chagas disease in pediatric population, and they reported that only 3 of 12 children in the acute phase presented with signs and symptoms of Chagas disease: low weight at birth, hepatosplenomegaly, respiratory distress, myocarditis, anaemia and jaundice.

Due to the usual asymptomatic course, it is especially important to develop screening programs for the early diagnosis of the disease.

During the first year of life, a positive serological test can result from the placental transfer of maternal antibodies. Therefore, the diagnosis should be based on methods for direct parasite detection (PCR or blood smear examination-microhaematocrit), to be able to start early treatment if a positive result is obtained. In addition, these tests are more sensitive at this period because the acute infection phase presents with greater parasitaemia [14]. For the patients diagnosed within this age range of our series, the diagnosis was carried out in 94% by parasitological tests.

In all cases in which PCR was performed on infected patients younger than one year-old, no false positives were identified (a positive serological test was confirmed after 9 months of age), in keeping with other studies [5]

For patients older than one year-old, like for adults, serology continues to be the most frequent method for diagnostic purposes.

Treatment during pediatric age is well tolerated and effective in 50–70% of cases, therefore it can decrease the risk of complications and future vertical transmission [17]. Benznidazole is the treatment of choice for CD (first treatment option in 98% of treated patients in our series), with nifurtimox being a normal second-line therapeutic option [7]. Benznidazole achieves cure for up to 100% of children with congenital infection treated during the first year of life and 76% in patients with acute infection, decreasing to 9–15% in adult patients with chronic disease [9]. Nifurtimox has cure rates of around 86% in children and 7–8% in adults [9].

Recently, a paediatric formulation of nifurtimox has been approved and it can probably will be used as a first line treatment as well in future.

The negativization of serology, and therefore cure in our series, is clearly greater in the group of patients diagnosed and treated within the first year of their life, in accordance with other studies (Rodriguez Guerineau et al, Simon et al). We highlight the low cure rate in those over than one year of age, probably due to the high median age at diagnosis (11.08 years). When the infection is not diagnosed in time, children enter the chronic phase of CD and they require a longer follow-up in time, in order to achieve cure criteria.

The main limitation in evaluating treatment response for CD (in patients older than 1 year, as in adults) stems from the need for a long-term follow-up (years to decades) to demonstrate seroreversion from positive antibodies to a negative serology with conventional tests. Alternative early markers of cure have been suggested, such as decrease of total anti T.cruzi antibody titers or ELISA anti F2/3[20], but nowadays the only criteria of CD cure is seroreversion [9].

In our case, of the 33 patients who continued follow-up, although only 9 patients (27,2%) were considered cured due to negative serology, 11 cases (33.3%) had decreasing antibody titers and will probably end up becoming negative when the follow up period will be prolonged.

Our results are similar to those of the series by Rodriguez-Guerineau et al, where they describe that the only factor associated with the cure rate was age at diagnosis and treatment (p = 0.008), finding a cure rate of 100% in children under one year, while it was only 4.2% in those over this age (median age: 4 years).

In other pediatric series published in endemic areas [21] the results of seroconversion were variable (from 5 to 90%), for different reasons (different parasite lineages, varying age groups and varying times of post-treatment follow-up and different times since acute phase of the disease).

There is better tolerance to treatment (lower AE rate) in children than in adults [22,23], specially when administered in patients younger than 7 years-old. In a study [24] conducted on 107 patients with CD treated with benznidazole, AE were described in 41% of cases, 77.3% of wich were in children older than 7 years-old.

In our series of patients, AE due to benznidazole were reported in almost 50% of cases, more frequent in children older than one year (78.2% of patients with benznidazole AE), with a median age of 11.4 years. Among the 32 patients older tan one year treated with benznidazole, 18 (56.25%) had adverse events whereas in the 15 under one year-old, 5(33,3%) did.

Our study presents some limitations, firstly due to its retrospective nature and because it presents a significant percentage of losses to follow-up (35%), higher than in other series in non-endemic areas [9], although this information is provided in few publications under the conditions of our study. We believe that this figure is largely attributable to the characteristics of the studied population (immigrant population that sometimes has difficulties in going to medical appointments or returns to their country of origin) [25]. This high rate of losses to follow-up is of concern and efforts should be made to improve it.

Another limitation could be the variability in the serological techniques among the different hospitals. Moreover, some hospitals changed the technique over time. Therefore, the ability to detect antibodies could be different and furthermore the new serological techniques detect lower levels of antibodies. This reinforces the need to explore new cure markers of this disease, more accurate than conventional serology.

The mean follow-up time has been heterogeneous depending on the patients year of diagnosis, specially for those with no cure criteria. Although in all there has been a minimum follow-up time, until at least two serologies have been performed. This represents another limitation of the study and may condition the results interpretation since probably some patients with declining serology titers end up fullfiling cure criteria by prolonging follow-up.

However, it provides data of one of the largest series of pediatric CD patients in non-endemic areas, reinforcing the importance of early diagnosis and treatment, to achieve higher cure rates, particularly in children under one year. We also highlight, the considerable rate of treatment AE in the pediatric population older than one year of our series, higher than in other studies [19].

Prospective studies with longer follow-up period, to determinate more accurately the characteristics of treatment response in pediatric age and future complications in adulthood are needed.
